# Novel insight into the distribution and dissemination of *Candidatus* Liberibacter asiaticus, the causal agent of citrus Huanglongbing

**DOI:** 10.1111/pbi.13753

**Published:** 2021-12-09

**Authors:** Qinglian Wang, Yunlong Xu, Xiaofen Yang, Jin Jia, Jiale Zhou, Jiwu Zeng, Xiang Yan, Jinxue Li, Jianqiang Yue, Jun Guo, Yi Yang, Changxiu Xia, Ni Hong, Guoping Wang, Shu‐ang Peng, Yongping Duan, John S. Hartung, Fang Ding

**Affiliations:** ^1^ Hubei Key Laboratory of Plant Pathology, College of Plant Science and Technology Huazhong Agricultural University Wuhan China; ^2^ Key Laboratory of Horticultural Plant Biology (Huazhong Agricultural University) Ministry of Education Wuhan China; ^3^ Fruit Tree Research Institute Guangdong Academy of Agricultural Science Guangzhou China; ^4^ Ganzhou Citrus Research Institute Ganzhou China; ^5^ Institute of Tropical and Subtropical Cash Crops Yunnan Academy of Agricultural Science Baoshan China; ^6^ Environment and Plant Protection Institute Chinese Academy of Tropical Agricultural Science Haikou China; ^7^ USDA ARS USHRL Fort Pierce FL USA; ^8^ USDA ARS MPPL Beltsville MD USA

**Keywords:** citrus, Huanglongbing, viable *C*Las, pollen, distribution, cross‐pollination, dissemination

Huanglongbing (HLB), also known as citrus greening, is the most severe pandemics in citrus in more than 50 countries in Asia, Africa, and America, causing serious economic losses worldwide (Gottwald,^2020^ 2020; Wang, 2019). The disease is associated with a phloem‐limited and fastidious member of the α‐proteobacteriacea. ‘*Candidatus* Liberibacter asiaticus’ (*C*Las) is the most prevalent strain. It was frequently detected in leaves, stems, and roots based on PCR detection, which recognized both living and dead cells. Yet only intact and viable *C*Las cells are potentially infectious and transmissible. Previous study showed 17 to 31% of *C*Las cells were considered viable in HLB symptomatic tissues (Trivedi *et al*., 2009). Whether viable *C*Las is present in floral organs remains unclear and the possibility that the embryo could be infected via pollen has not yet been addressed. In this study, we aimed to identify viable *C*Las in citrus floral parts and the possibility of the dissemination through pollination process with anti‐OmpA and anti‐SDE1, two highly specific antibodies against *C*Las (Ding *et al*., 2020; Tran *et al*., 2020). These results will deepen our knowledge of the distribution *in planta* and the new dissemination pathway of *C*Las, which is important for the monitoring of HLB.

The study was carried out with 1113 samples of 5 citrus varieties collected in China. *C*Las DNA was detected in anther filament, pollen grains, stigma, ovary, and receptacle (Figure 1A). Significant differences were observed among the detection ratio in different floral organs (*P* < 0.05). Direct tissue blot immuno assay (DTBIA) revealed purple colour in the tissues from *C*Las‐infected stigmas, ovary, locules, and receptacle (Figure 1B). And in developing anthers, pollen grains, and the germinated pollen tubes (Figure 1C).

**Figure 1:**
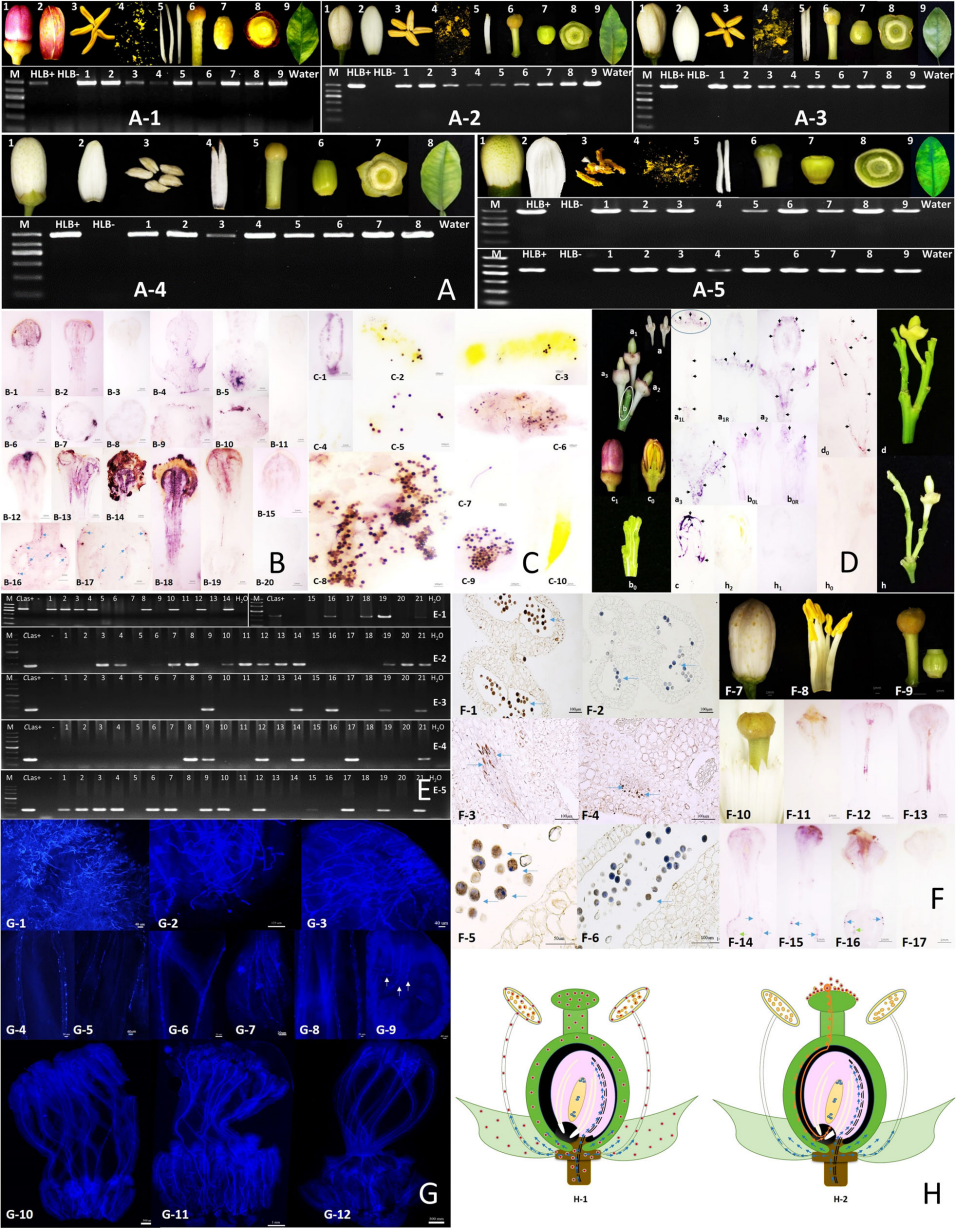
Distribution and dissemination of *C*Las through floral organs. (A) Detection of *C*Las in *Citrus limon* from Yunnan (A‐1), Green orange (*C*. *sinensis*) from Hainan (A‐2), Sweet orange (*C. sinensis*) (A‐3), Newhall navel orange (*C*. *sinensis*) (A‐4), and Wendan pummelo (*C. grandis*) (A‐5) from Jiangxi (the smaller band was reconfirmed by nested‐PCR). (B) Identification of viable *C*Las in floral organs before (B‐1 to B‐11) and after pollination (B‐12 to B‐20). (C) Identification of viable *C*Las in anthers and pollen grains. Signal was observed in immature anther walls (C‐1), partially mature (C‐2 to C‐5, except C‐4), and fully mature (C‐6) anthers. And from the entire pollen grains (C‐8 and C‐9) and pollen tubes (C‐6 and C‐7). (D) Invasion of flowers. Viable *C*Las in peduncle (a_1_‐a_3_), receptacle (a_1_, a_2_) and ovary (a_2_); and unopened flower bud (c_0_, c_1_). ‘b_0L_’, ‘b_0R_’, and ‘d_0_’ showed the distribution of *C*Las in the branches with pedicels. (E) Detection of *C*Las before and after controlled‐pollination. (E‐1) PCR detection of *C*Las from paternal pollens with OI1/OI2c (left) and CGO (right) primer. (E‐2) Nested‐PCR detection of *C*Las in stigma at 3 dpp (Lane1‐10), 5 dpp (Lane 11‐18), 10 dpp (Lane 19‐21), in stylet and ovary at 3 dpp (Lane 1‐4, E‐3), 5 dpp (Lane 5‐12, E‐3), 10 dpp (Lane 13‐21, E‐3), 15 dpp (Lane 1‐21, E‐4), and 30 dpp (lane1‐21, E‐5) after cross‐pollination. (F) Immuno histochemical analysis of *C*Las‐affected pollen from paternal parent. Positive signals in the locules (F‐1) and in vascular bundles (F‐3 and F‐4). F‐5 and F‐6 were higher magnifications. After cross‐pollination, signals were observed on the stigma in 4 hpp (F‐11), and in style in 2 dpp (F‐12 and F‐13) as well as in ovary and locules indicated by arrows in 3 dpp (F‐14 to F‐16). (G) Germination and growth of pollen tubes. Germinated pollen grains on stigma in 2 dpp (G‐1), 3 dpp (G‐2), and 5 dpp (G‐3). Pollen tube extended to stylet and ovary in 2 dpp (G‐4, G‐5, and G‐10), 3 dpi (G‐6, G‐7, and G‐11), and 5 dpi (G‐8, G‐9, and G‐12). (H) Schematic diagram showing the distribution of *C*Las in citrus floral parts (H‐1) and the route of infection of the ovary (H‐2). Red circles represent *C*Las bacteria. Orange arrows in ‘H‐2’ represent the movement of *C*Las within germinating pollen. Blue arrows represent movement of *C*Las from maternal plant.

We expected that the invasion of viable *C*Las into floral parts would follow the same as the invasion of branch buds. Unpollinated lemon flower buds were tested. *C*Las was located in the phloem of both the branch and flower bud, especially in the joint connection between the receptacle and flower branch (Figure 1D). Therefore, the early infection of flower buds was originated from the mother plant through phloem connections.

To find out the possibility of *C*Las dissemination from stigma to ovary, artificial cross‐pollination was performed (Figure 1F). In paternal pollens, signals were observed inside the pollen grains in the locules as well as in the vascular bundles. After cross‐pollination, *C*Las were firstly detected on the stigma in 4 hpp, and then moved from stigma to stylet via pollen tubes. In 3 dpp, *C*Las was found in the ovary and mainly localized in the ovary wall, as well as in locules. These results coincided with PCR (Figure 1E) and were consistent with the germination rate of *C*Las‐affected pollen tubes observed by fluorescence microscopy (Figure 1G). Our results showed the dissemination of *C*Las from stigma to ovary through pollination process.

In this study, the presence of viable *C*Las in the pollen itself, especially in the pollen tube, opens the possibility that infection of the embryo could occur from the pollen and thus avoid the chalazal barrier. Based on our data, the distribution of *C*Las within an infected flower and a pathway for the potential infection of the ovary of a healthy flower can be summarized (Figure 1H). It is worth noting that seedlings grew out from *C*Las‐affected fertile seeds indicated the existence of the bacteria in extreme low titre and became undetectable in the late stage (data not shown). We suggest that the aborted seed may be the result of infection of the embryo via pollination, whereas the viable seed with contaminated seed coats or rarely embryo are largely due to the infection from the maternal plant. The low title of *C*Las infection failed to sustain itself as the seedlings grew, suggesting that the *C*Las populations being transmitted to the seedling either not existed in a viable form or missed some of the populations necessary for multiplication and virulence. In conclusion, viable *C*Las was firstly identified in citrus floral organs, especially in pollen and pollen tubes. The distribution of *C*Las in pollen grains and pollen tubes opened a new possible dissemination pathway through pollination which should be also taken into account for the integrated control of HLB.

## Conflict of interest

The authors declare no competing interests.

## Author contributions

Q.L. Wang, Y.L. Xu, and X.F. Yang initiated the study, and contributed equally; J. Jia, J.L. Zhou, J.W. Zeng, X. Yan, J.X. Li, J.Q Yue, J. Guo, Y. Yang, C.X. Xia contributed samples and data analysis. N. Hong, G.P. Wang, S.A. Peng, Y.P. Duan, J.S. Hartung, and F. Ding contributed to critically revising of the manuscript.
